# Low‐dose gemcitabine plus nab‐paclitaxel *versus* standard‐dose gemcitabine plus nab‐paclitaxel in elderly patients with metastatic pancreatic cancer: A randomized Phase II trial

**DOI:** 10.1002/jgh3.12966

**Published:** 2023-09-07

**Authors:** Ken Kamata, Hajime Imai, Hisakazu Matsumoto, Yukitaka Yamashita, Takao Kato, Katsuhisa Nishi, Shunsuke Omoto, Kosuke Minaga, Kentaro Yamao, Tomoko Hyodo, Sung‐Woon Im, Akane Hara, Tomoe Yoshikawa, Rei Ishikawa, Ayana Okamoto, Tomohiro Yamazaki, Atsushi Nakai, Kazuomi Ueshima, Yasutaka Chiba, Mamoru Takenaka, Tomohiro Watanabe, Masayuki Kitano, Masatoshi Kudo

**Affiliations:** ^1^ Department of Gastroenterology and Hepatology Kindai University Faculty of Medicine Osaka Japan; ^2^ Department of Gastroenterology Okanami General Hospital Mie Japan; ^3^ Department of Gastroenterology Japanese Red Cross Wakayama Medical Center Wakayama Japan; ^4^ Department of Gastroenterology Hyogo Prefectural Awaji Medical Center Hyogo Japan; ^5^ Department of Radiology Kindai University Faculty of Medicine Osaka‐Sayama Japan; ^6^ Clinical Research Center Kindai University Hospital Osaka Japan; ^7^ Second Department of Internal Medicine Wakayama Medical University School of Medicine Wakayama Japan

**Keywords:** chemotherapy, elderly, gemcitabine, nab‐paclitaxel, pancreatic cancer

## Abstract

**Background and Aim:**

A multicenter, open‐label randomized Phase II trial was conducted to determine whether low‐dose gemcitabine plus nab‐paclitaxel (GnP) could improve tolerability and show equivalent efficacy to the standard‐dose GnP for elderly patients with metastatic pancreatic cancer.

**Methods:**

Consecutive patients aged ≥65 years with metastatic pancreatic cancer who presented at one of four Japanese referral centers between November 2016 and January 2021 were enrolled. The 60 patients were randomly assigned to low‐ or standard‐dose groups with a 1:1 ratio. Patients in the low‐dose GnP group received gemcitabine at a dose of 250 mg/m^2^ and nab‐paclitaxel at 125 mg/m^2^.

**Results:**

Low‐dose GnP significantly decreased the rate of cases requiring dose reduction (16.7% *vs* 63.3%). The response rate (36.7% *vs* 33.3%) and progression‐free survival (7.3 *vs* 8 months) were comparable between the low‐ and standard‐dose groups as determined by independent review. The difference in the median overall survival between the two groups was not significant (7.9 *vs* 12 months). The proportion of patients with hematologic and non‐hematologic treatment‐related adverse events was comparable between the two groups.

**Conclusion:**

Low‐dose GnP had an equivalent efficacy to conventional therapy; however, it did not reduce adverse events.

## Graphical abstract

The response rate (36.7% *vs* 33.3%) and progression‐free survival (7.3 *vs* 8 months) were comparable between the low‐ and standard‐dose gemcitabine plus nab‐paclitaxel groups as determined by independent review.

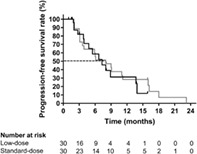

## Introduction

Gemcitabine plus nab‐paclitaxel (GnP) therapy is widely used as a standard therapy for patients with metastatic pancreatic cancer after the verification of an overall survival advantage of this regimen over gemcitabine (GEM) monotherapy in the MPACT study.^1^ The response rates in the GnP and GEM monotherapy groups against metastatic pancreatic cancer were 23 and 7%, respectively.^1^ Given that response rates of GEM monotherapy are <10% in clinical trials^2^, ^3^ including the MPACT study, much progress has been achieved in GnP therapy. In a previous study, tumor tissues from 11 patients with pancreatic cancer were transplanted subcutaneously into nude mice, which were divided into three groups: GEM monotherapy, nab‐paclitaxel (nab‐PTX) monotherapy, and GnP.^4^ The tumor reduction rates in the three groups were 24, 36 and 55%, respectively. Collectively, it is suggested that the improvement in the response rate to GnP therapy is largely due to the contribution of nab‐PTX. Regarding safety of GnP, a domestic phase I/II study reported that the incidence rates of grade 3 or severe leukopenia and neutropenia in patients treated with GnP were 52.9 and 67.6%, respectively.^5^ Thus, suppression of bone marrow function is one of the major concerns associated with GnP. In this regard, the use of GnP requires particular caution in elderly patients since turnover of hematopoietic stem cells is slower in the elderly than younger patients. Although the World Health Organization defines the elderly as those over 65 years of age, it remains largely unknown low doses regimens need to be applied in such elderly patients to avoid adverse events associated with chemotherapies.^6^ Parallel to the GnP therapy, FOLFIRINOX is recommended as first‐line treatment for metastatic pancreatic cancer; however, the use of this therapy is limited in patients ≥65 years of age due to its high toxicity.^3^ To address the safety and efficacy of the GnP therapy for the elderly patients (≥65 years old) bearing metastatic pancreatic cancer, we performed the randomized Phase II trial. We randomly divided the elderly patients with metastatic pancreatic cancer into two groups: the standard‐dose GnP therapy and low‐dose GnP therapy. The low‐dose GnP therapy was consisted of a reduced‐dose regimen of GEM and standard‐dose of nab‐PTX. This trial was conducted to determine whether low‐dose GnP could improve tolerability and have an equivalent efficacy to standard‐dose GnP for elderly patients with metastatic pancreatic cancer.

## Methods

### Study design

This multicenter, open‐label randomized Phase II trial was approved by the *Ethics Committee* of the Kindai University Faculty of Medicine (number: 28‐114). All the patients provided written informed consent. The trial was registered with the University Hospital Medical Information Network (UMIN000024505, date of the first registration: 20 October 2016). All methods were performed in accordance with the relevant guidelines and regulations. The included patients were randomly assigned to two groups with a 1:1 ratio using a random number generator. One group received low‐dose GnP and the other group received standard‐dose GnP. Patients were stratified according to performance status. Treatment continued until disease progression or until appearance of severe adverse events occurred. There was no provision for second‐line treatment and beyond. Patients were followed up to evaluate the clinical outcome until death or study closure (2 years after the registration ends).

### Patients

Consecutive patients with metastatic pancreatic cancer who presented at one of four Japanese referral centers between November 2016 and January 2021 were prospectively enrolled. Patients were included if they were ≥65 years of age, had a performance status ≤1, had histologically or cytologically confirmed adenocarcinoma of the pancreas, and had provided written informed consent. Eligible patients included those treated with adjuvant or neoadjuvant therapy if they received treatment at least 6 months before randomization. Patients were excluded if they had pulmonary fibrosis, interstitial pneumonia, active infection, symptomatic brain metastasis, active multiple cancers, or severe mental disorders. Adequate hematologic, hepatic, and renal function (including an absolute neutrophil count of ≥1500/m^2^, a hemoglobin level of ≥9 g/deciliter, and a platelet count of ≥100 000/m^2^) was required.

### Treatment regimen

Patients in the low‐dose GnP group received infusion of GEM at a dose of 250 mg/m^2^ and infusion of nab‐PTX at a dose of 125 mg/m^2^, on days 1, 8, and 15 every 4 weeks. Patients in the standard‐dose GnP group received infusion of GEM at a dose of 1000 mg/m^2^ and infusion of nab‐PTX at a dose of 125 mg/m^2^, on days 1, 8, and 15 every 4 weeks. In the low‐dose GnP treatment regimen, the dose reduction applied only to nab‐PTX. The rationale for setting the dosage of low‐dose GEM in the present study was as follows. In the MPACT study, the relative dose intensity for GEM in standard‐dose GnP therapy and standard‐dose GEM monotherapy was 75 and 85%, respectively.^1^ However, the median cumulative dose of GEM was 11 400 mg/m^2^ and 9000 mg/m^2^ for standard‐dose GnP therapy and standard‐dose GEM monotherapy, respectively. Thus, we hypothesized that even if the relative dose intensity of GEM in GnP therapy was reduced, the cumulative dose necessary for tumor reduction could be achieved by extending the administration period. GEM is phosphorylated intracellularly and is metabolized to triphosphate (dFdCTP),^7^ an inhibitor of DNA synthesis. In a phase I study of GEM monotherapy, the intracellular concentration of dFdCTP increased in a dose‐proportional manner at doses of 35–250 mg; however, at doses of 350 mg/m^2^ or higher, the dFdCTP concentration became saturated.^7^ Based on this report, we previously conducted a small comparative study of standard‐dose GEM monotherapy and low‐dose GEM monotherapy (250 mg/m^2^) in patients with unresectable pancreatic cancer and found that low‐dose GEM had similar efficacy and a lower incidence of adverse events than standard‐dose GEM.^8^ Thus, we assume that low‐dose GEM (250 mg/m^2^) was effective when administered together with standard‐dose nab‐PTX.

### Outcome measures

The primary endpoint was the response rate. The secondary endpoints were overall survival, progression‐free survival, disease control rate, and adverse event rate. The response rate, progression‐free survival, and disease control rate were analyzed by investigators and an independent review committee according to the RECIST, version 1.1. The tumor response was evaluated by computed tomography performed every 6 weeks. Complete and partial responses required confirmation of response after 6 weeks. Response rate was defined as the percentage of patients with a complete response or partial response for the best overall response. Disease control rate was defined as the percentage of patients with a complete response, partial response, or stable disease for the best overall response. The grade of the treatment‐related adverse events was assessed according to the National Cancer Institute Common Terminology Criteria for Adverse Events, version 4.0.

### Statistical analysis

Continuous and categorical variables were analyzed using *t* tests and chi‐square tests, respectively. Response rates and disease control rates for each group were calculated with 95% confidence intervals (CIs) and compared using the chi‐square test. Progression‐free survival and overall survival were defined as the time from registration until disease progression or death. Median survival times with 95% CIs were estimated by the Kaplan–Meier method and compared with the log‐rank test. Data were censored if the patients did not have an event by the time of the analysis or if they were lost to follow‐up. In a Japanese Phase II study, the response rate to GnP in patients with metastatic pancreatic cancer was 58.8% (95% CI: 40.7–75.4).^5^ In the international phase III study (MPACT study), it was 23% (95% CI: 19–27%).^1^ The expected response rate for low‐dose GnP in the present study was 40%, and the expected threshold efficacy rate was 15%. Under this assumption, a type I error of 0.05 (one‐sided), a power of 90%, and a sample of 30 patients in one group would be required, assuming a certain drop‐out rate. If the results were obtained as hypothesized, an overall evaluation was made with reference to the secondary endpoints. All statistical analyses were performed using GraphPad Prism 6.0 software (GraphPad Software Inc., San Diego, CA, USA).

## Results

### Patient characteristics

A total of 60 patients with metastatic pancreatic cancer were enrolled. The median age was 73 (range, 65–87) years. Of the 60 patients, 30 were randomized into the low‐dose GnP group and 30 into the standard‐dose GnP group. In eligible patients, postoperative metastatic recurrence included three patients in the low‐dose group and four patients in the standard‐dose group. One patient in the low‐dose group withdrew consent and did not receive treatment. The remaining 59 patients received the study treatment. The groups did not differ significantly in terms of demographic and clinical characteristics at baseline (Table 1).

**Table 1:** Patient characteristics at baseline

Characteristics	Low‐dose (*n* = 30)	Standard‐dose (*n* = 30)	Total (*n* = 60)	*P* value^†^
Age, *n*				
<75	19	19	38	1.000
≥75	11	11	22	
Sex, *n*				
Female	15	15	30	1.000
Male	15	15	30	
Performance status, *n*				
0	30	29	59	1.000
1	0	1	1	
Tumor location, *n*				
Head	12	11	23	1.000
Body or tail	18	19	37	
Metastatic site, *n* ^‡^				
Liver	21	23	44	0.771
Lung	6	8	14	0.761
Lymph node	9	6	15	0.552
Peritoneum	3	6	9	0.472
Pleura	0	1	1	1.000
Number of metastatic sites, *n*				
1	22	20	42	0.779
2	7	6	13	1.000
3	1	4	5	0.353
>3	0	0	0	1.000
Median carbohydrate antigen 19–9, U/ml^§^	561	780	774	0.746
Previous therapy, *n*				
Pancreatectomy	3	4	7	1.000
Biliary stent	5	7	12	0.748

^†^ Compared between low‐ and standard‐dose groups using Mann–Whitney test for median and chi‐squared test for categorical variables.

^‡^ Overlap included.

^§^ Calculated as 3 U/ml if not measurable due to less than 3 U/ml. Data were missing in one patient in the low‐dose group.

### Treatment status

The treatment status is shown in Table 2. Although one patient who withdrew consent in the low‐dose group was excluded from analysis of the median relative dose intensity (the percentage of the administered cumulative dose relative to the planned cumulative dose), other values were examined in all 60 patients. The median duration of the protocol treatment was 3.2 months (range, 0.1–14.9 months) in the low‐dose group and 5.1 months (range, 0.1–15.6 months) in the standard‐dose group; 26.7 and 40.0% of patients received treatment for at least 6 months, respectively. The main reasons for discontinuation of treatment were progressive disease and poor tolerance in both groups. The median relative dose intensity in the low‐dose group was 86.0% for nab‐PTX and 90% for GEM, whereas that in the standard‐dose group was 76.0% for nab‐PTX and 76.5% for GEM. The rate of use of subsequent treatment was balanced between the two groups: 36.7% for the low‐dose group and 43.3% for the standard‐dose group. In the overall statistical evaluation, the groups differed significantly in terms of median relative dose intensity for GEM and the number of cases requiring dose reduction. The mean skipped doses number was higher in the standard‐dose group than in the low‐dose group. No significant differences or trends were found in other treatment characteristics.

**Table 2:** Treatment status^†^

	Low‐dose (*n* = 30)	Standard‐dose (*n* = 30)	*P* value^†^
Median duration of protocol treatment, months	3.2 (0.1–14.9)	5.1 (0.1–15.6)	0.199
Receiving protocol treatment for at least 6 months, *n* (%)	8 (26.7)	12 (40.0)	0.412
Receiving protocol treatment for less than 2 months, *n* (%)	8 (26.7)	6 (20.0)	0.532
Reason for discontinuation of protocol treatment, *n*			
Progressive disease^‡^	10	9	1.000
Poor tolerance	12	11	1.000
Reduced performance status	5	7	0.748
Cause‐specific death	0	1	1.000
Others^§^	3	2	1.000
Median relative dose intensity, %^¶^			
nab‐PTX	90.0	76.5	0.304
GEM	86.0	76.0	0.008
Mean number of skipped doses, *n* (SD)	1.8 ± 2.3	3.6 ± 4.5	0.056
Number of cases requiring dose reduction, *n* (%)	5 (16.7)	19 (63.3)	<0.001
Second‐line therapy, *n*			
TS‐1	7	10	0.567
nal‐IRI + 5FU/LV	3	3	1.000
FOLFIRINOX	1	0	1.000

^†^ Compared between low‐ and standard‐dose groups using t test for mean, Mann–Whitney test for median, and chi‐squared test for categorical variables.

^‡^ Judgment by investigators.

^§^ Including cases in which the next course could not be started for more than 28 days after the last day of the treatment because of comorbidities such as cholangitis, or cases in which continuous treatment was difficult because of relocation, hospital transfer, or busy schedule.

^¶^ One patient in the low‐dose group refused to start treatment; therefore, values were calculated after excluding this patient.

GEM, gemcitabine; nab‐PTX, nab‐paclitaxel.

### Efficacy

#### Response

**Figure 1:**
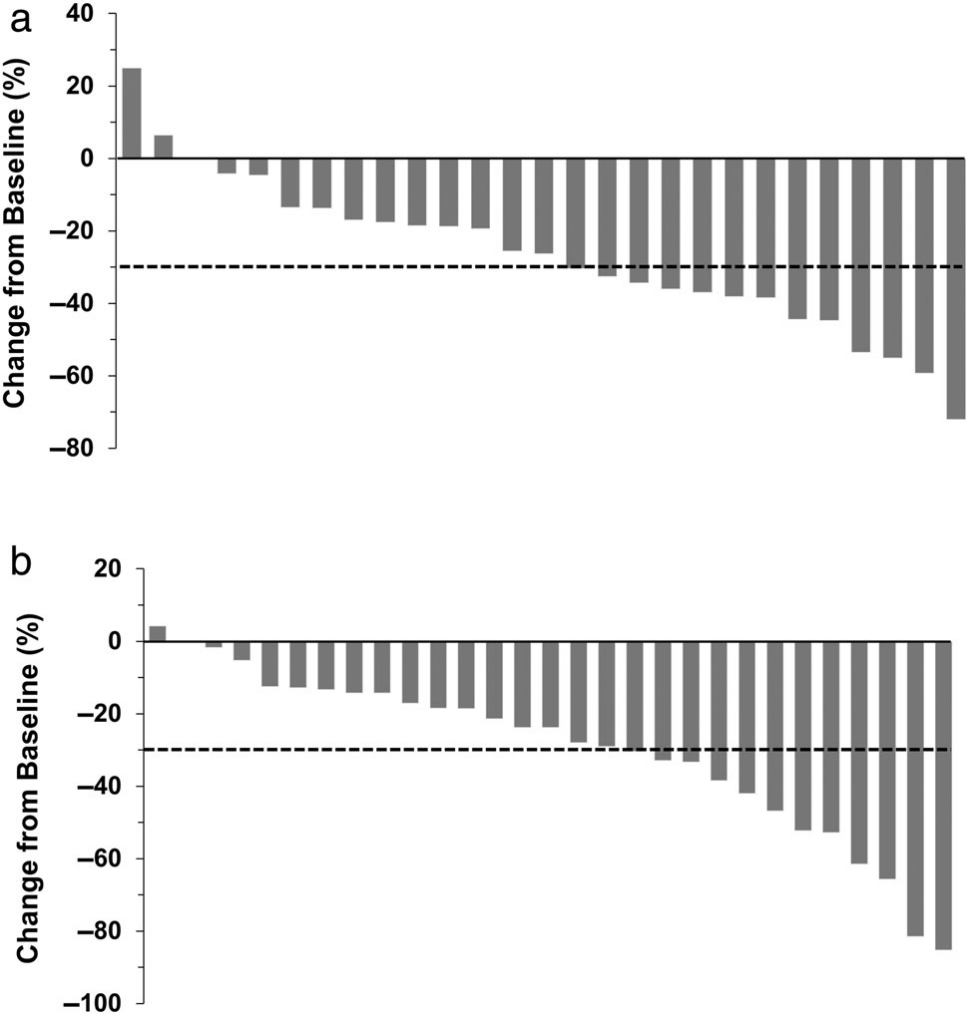
Waterfall plots indicating tumor response according to independent review in the low‐dose group (a) and standard‐dose group (b).

**Figure 2:**
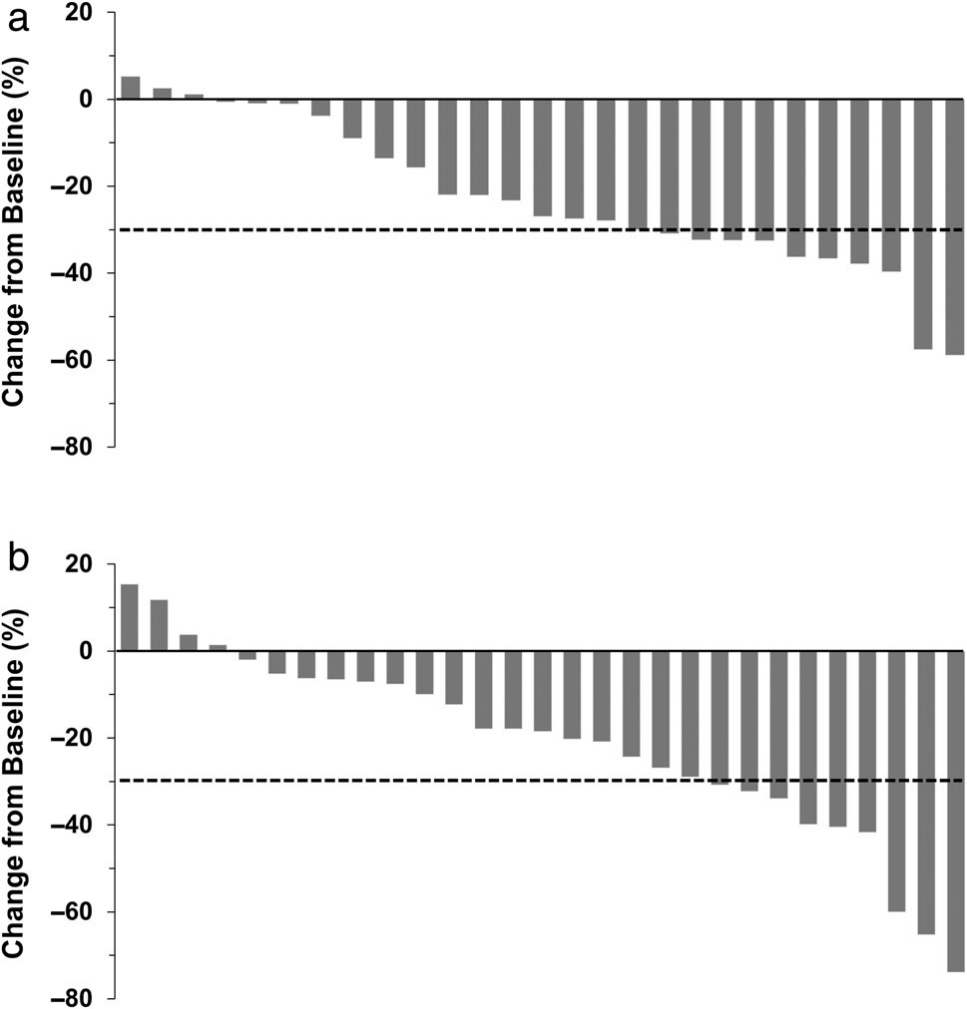
Waterfall plot indicating tumor response according to investigator assessment in the low‐dose group (a) and standard‐dose group (b).

In the intention‐to‐treat population, the response rate according to independent review was comparable between the two groups (36.7% [95% CI: 21.8–54.5] *vs* 33.3% [95% CI: 19.1–51.3]; *P* = 1.000; odds ratio for response rate, 1.16 [95% CI: 0.41–3.30]) (Table 3). The waterfall plot showed that the maximum percentage of change was higher in the standard‐dose group than in the low‐dose group, although the number of patients with reduction of ≥30% was higher in the low‐dose group than in the standard‐dose group (Fig. 1). The disease control rate according to independent review was comparable between the two groups (76.7% [95% CI: 58.8–88.5] *vs* 83.3% [95% CI: 66.0–93.1]; *P* = 0.541; odds ratio for disease control rate, 0.66 [95% CI: 0.19–2.27]). Investigator assessment of the response rate and disease control rate showed similar results to those obtained by independent review (Fig. 2 and Table 3).

**Table 3:** Response rate, overall survival, progression‐free survival, and disease control rate in the intention‐to‐treat population

	Low‐dose (*n* = 30)	Standard‐dose (*n* = 30)	Hazard ratio or odds ratio (95% CI)^†^	*P* value
Response assessed by independent review committee				
Response rate, % (95% CI)	36.7 (21.8–54.5)	33.3 (19.1–51.3)	1.16 (0.41–3.30)	1.000
Disease control rate, % (95% CI)	76.7 (58.8–88.5)	83.3 (66.0–93.1)	0.66 (0.19–2.27)	0.541
Best response, *n*				
Complete response	0	0		
Partial response	11	10		
Stable disease	12	15		
Progressive disease	4	4		
Not evaluated	3	1		
Response assessed by investigators				
Response rate, % (95% CI)	33.3 (19.1–51.3)	26.7 (14.0–44.7)	1.38 (0.46–4.08)	0.779
Disease control rate, % (95% CI)	76.7 (58.8–88.5)	86.7 (69.7–95.3)	0.51 (0.14–1.85)	0.343
Best response, *n*				
Complete response	0	0		
Partial response	10	8		
Stable disease	13	18		
Progressive disease	4	3		
Not evaluated	3	1		
Progression‐free survival assessed by independent review committee				
Median progression‐free survival, month (95% CI)	7.3 (3.9–13.7)	8.0 (3.6–11.2)	1.20 (0.60–2.39)	0.582
Progression‐free survival rate, % (95% CI)				
6 months	58.6 (33.2–77.2)	55.7 (34.3–72.7)		
12 months	31.3 (11.0–54.2)	28.3 (11.9–47.3)		
Progression‐free survival assessed by investigators				
Median progression‐free survival, month (95% CI)	6.7 (4.8–11.0)	7.4 (4.2–11.0)	1.19 (0.63–2.23)	0.570
Progression‐free survival rate, % (95% CI)				
6 months	53.1 (29.8–71.8)	61.3 (40.0–77.1)		
12 months	24.3 (8.0–45.3)	20.4 (7.5–37.8)		
Overall survival				
Median overall survival, month (95% CI)	7.9 (4.8–11.1)	12.0 (5.9–15.4)	1.33 (0.79–2.26)	0.271
Survival rate, % (95% CI)				
6 months	66.7 (46.9–80.5)	66.7 (46.9–80.5)		
12 months	26.7 (12.6–43.0)	50.0 (31.3–66.1)		
18 months	13.3 (4.2–27.8)	22.9 (9.9–39.1)		
24 months	6.7 (1.2–19.2)	14.3 (4.2–30.2)		

^†^ Odds ratio was used for response and disease control rate. Hazard ratio was used for progression‐free survival rate and overall survival. Hazard ratio <1 and odds ratio >1 indicate a favorable low‐dose group.

CI, confidence interval.

#### Progression‐free survival

**Figure 3:**
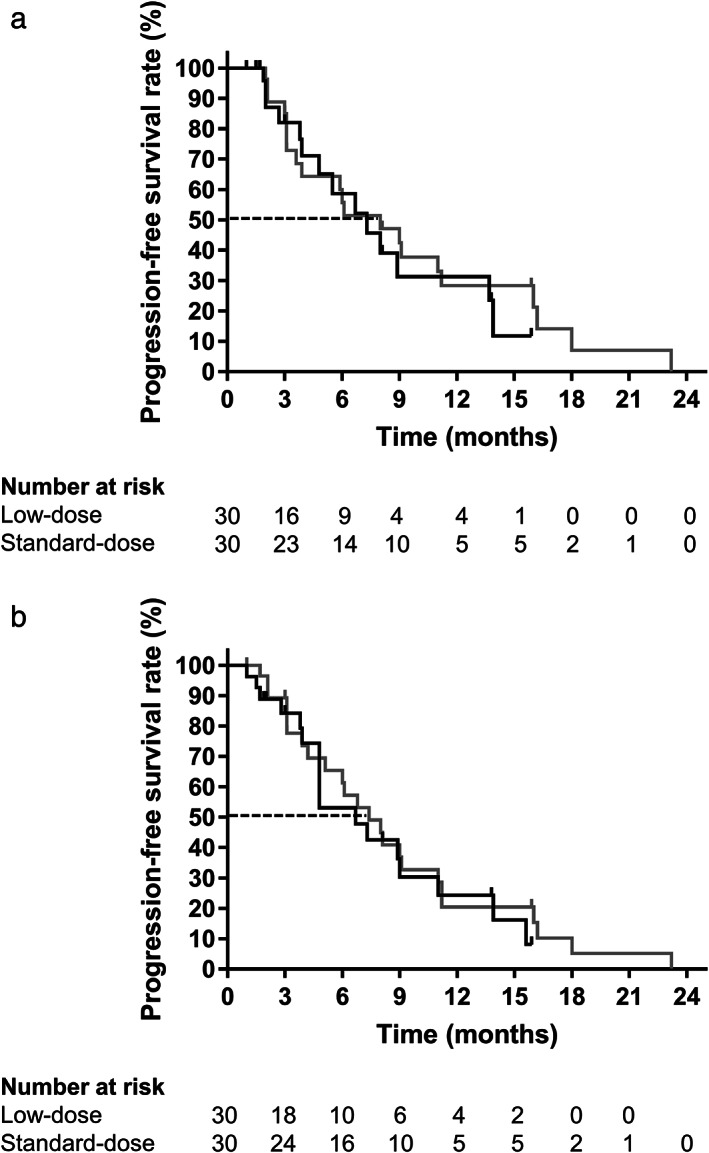
Progression‐free survival curves of low‐ and standard‐dose groups according to independent review (a) and investigator assessment (b). (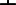), Low‐dose; (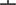), standard‐dose.

In the assessment of progression‐free survival according to independent review, 14 patients (46.7%) had progression of disease or died in the low‐dose group compared with 21 patients (70%) in the standard‐dose group. The progression‐free survival based on independent review was comparable between the two groups (7.3 months [95% CI: 3.9–13.7] *vs* 8 months [95% CI: 3.6–11.2]; *P* = 0.582; hazard ratio for disease progression or death, 1.20 [95% CI: 0.60–2.39]) (Fig. 3a and Table 3). The 6‐month and 1‐year progression‐free survival rates were 58.6 and 31.3% in the low‐dose group, whereas those in the standard‐dose group were 55.7 and 28.3%, respectively. Investigator assessment of progression‐free survival showed similar results to those obtained by independent review (Fig. 3b and Table 3).

#### Overall survival

**Figure 4:**
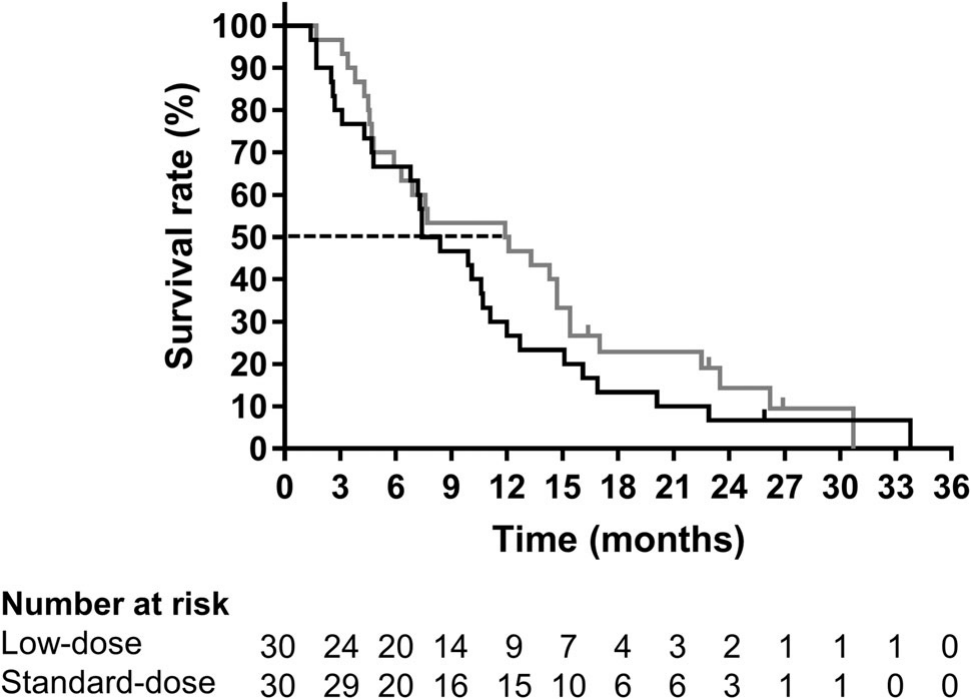
Overall survival curves of low‐ and standard‐dose groups. (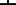), Low‐dose; (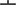), standard‐dose

In the assessment of overall survival, 29 patients (96.7%) died in the low‐dose group, whereas 27 patients (90.0%) died in the standard‐dose group. The median overall survival was higher in the standard‐dose group than in the low‐dose group, although the difference was not significant (7.9 months [95% CI: 4.8–11.1] *vs* 12 months [95% CI: 5.9–15.4]; *P* = 0.271; hazard ratio for death, 1.33 [95% CI: 0.79–2.26]) (Fig. 4 and Table 3). The 6‐month and 1‐year survival rates were 66.7 and 26.7% in the low‐dose group, whereas those in the standard group were 66.7 and 50.0%, respectively.

### Safety

The proportion of patients with hematologic and non‐hematologic treatment‐related adverse events was comparable between the two groups (Table 4). No fatal events related to the treatment were observed. Febrile neutropenia occurred in one case (3.3%) in each group. The most common grade 3 or higher hematologic adverse events were neutropenia (50.0% in the low‐dose group and 56.7% in the standard‐dose group) and leukopenia (40.0% in the low‐dose group and 36.7% in the standard‐dose group). The median time from initial treatment to the occurrence of grade 3 or higher hematologic adverse events was 14 days in both groups. Thus, grade 3 or higher hematologic toxicity and time to onset were comparable between the two groups (Table 4). Furthermore, the frequency of grade 3 or higher hematologic toxicity in patients aged 75 years and older was similar between the low‐dose group (63.6% [7/11]) and the standard‐dose group (54.5% [6/11]). All patients were administered dexamethasone and palonosetron as premedication. The most frequent non‐hematologic adverse events were alopecia (90.0% in the low‐dose group and 86.7% in the standard‐dose group) and peripheral neuropathy (80.0% in the low‐dose group and 76.7% in the standard‐dose group). There were fewer than two patients with grade 3 or higher non‐hematologic adverse events in either group. Interstitial pneumonia was observed in one case (3.3%) in each group. Serious adverse events occurred in 11 patients (36.7%) in the low‐dose group and in 12 patients (40.0%) in the standard‐dose group.

**Table 4:** Treatment‐related adverse events based on the investigator assessment and use of growth factors

	Low‐dose (*n* = 30)		Standard‐dose (*n* = 30)		*P* value	
Adverse events leading to death, *n* (%)	0 (0)		0 (0)		1.000	
Febrile neutropenia, *n* (%)	1 (3.3)		1 (3.3)		1.000	
Treatment with growth factors, *n* (%)	1 (3.3)		2 (6.6)		1.000	
	Grade ≥ 3	Any grade	Grade ≥ 3	Any grade	Grade ≥ 3	Any grade
Hematologic adverse events, *n* (%)						
Neutropenia	15 (50.0)	21 (70.0)	17 (56.7)	21 (70.0)	0.617	1.000
Leukopenia	12 (40.0)	20 (66.7)	11 (36.7)	21 (70.0)	1.000	1.000
Thrombocytopenia	6 (20)	10 (33.3)	7 (23.3)	10 (33.3)	1.000	1.000
Anemia^†^	1 (3.3)	23 (76.7)	2 (6.7)	24 (80.0)	1.000	1.000
Non‐hematologic adverse events occurring in at least 30% of patients or grade 3 or higher occurring in at least 5% of patients, *n* (%)						
Alopecia	NA	27 (90.0)	NA	26 (86.7)	NA	1.000
Peripheral neuropathy	0 (0)	24 (80.0)	1 (3.3)	23 (76.7)	1.000	1.000
Fatigue	1 (3.3)	18 (60.0)	1 (3.3)	18 (60.0)	1.000	1.000
Appetite loss	2 (6.6)	9 (30.0)	1 (3.3)	12 (40.0)	1.000	0.589
Nausea	2 (6.6)	9 (30.0)	1 (3.3)	8 (26.7)	1.000	1.000
Diarrhea	0 (0)	5 (16.7)	2 (6.6)	7 (23.3)	0.492	0.748

^†^ For Grade 1 and 2, the counts were assumed to be lower than the baseline values.

NA, not applicable.

## Discussion

According to the International Agency for Research on Cancer database for 2008–2012, the age‐standardized incidence rates for pancreatic cancer in major cities around the world are higher in patients aged ≥60 years than in those aged <59 years.^9^ Consistent with these age‐standardized incident rates, the mean age of patients with pancreatic cancer is over 60 in two major trials.^1^, ^3^ In the ACCORD11 trial published in 2011, which showed the superiority of FOLFIRINOX over GEM monotherapy for patients with unresectable pancreatic cancer, the median age of the 342 eligible patients was 61 years.^3^ The median age of the 861 eligible patients in the MPACT study published in 2013 was 63 years, and 42% of all patients were 65 years or older.^1^ These findings suggest that the age of onset of pancreatic cancer is expected to increase in the future. The median age of the patients in the present study was 73 years, and 36.7% of the enrolled patients were older than 75 years. Such elderly patients are considered vulnerable to side effects of anti‐cancer agents.^6^ In this randomized Phase II study, the elderly patients with metastatic pancreatic cancer exhibited comparable rates of adverse events as well as efficacy whether they were treated with standard‐dose GnP or low‐dose GnP. Therefore, our study provides direct evidence that reduction in GEM doses is not beneficial for the elderly patients bearing metastatic pancreatic cancer. However, low‐dose GnP regimen applied in this trial might be beneficial for burden of medical expenses.

Five trials have addressed safety and efficacy of GnP therapy in the elderly patients with pancreatic cancer.^10^, ^11^, ^12^, ^13^, ^14^ Rehman *et al*. conducted a retrospective study of the elderly 73 patients (over 65 years old) with advanced pancreatic cancer who received an attenuated regimen of biweekly GEM 1000 mg/m^2^ and nab‐PTX 125 mg/m^2^, that is, another low‐dose GnP regimen. The results showed that the median overall survival and progression‐free survival were 9.1 and 4.8 months, respectively, both of which parameters for the evaluation of efficacy were not inferior to those reported in the MPACT study.^10^ Moreover, the incidence of grade 3 or higher toxicities on neutropenia, anemia, thrombocytopenia, and neurotoxicity was reduced to 2, 7, 3, and 5%, respectively. Such low incidences of bone marrow suppression were associated with relatively low rates in patients who were treated with reduced dose with GEM (10%) and nab‐PTX (4%). In contrast, in the present study, adverse events associated with bone marrow function were comparable between the elderly patients treated with low‐ and standard‐dose GnP. These conflicting data can be partially explained by the finding that nab‐PTX rather than GnP might be toxic to bone marrow function in this regimen (see further discussion below). The other four trials showed that standard‐dose GnP is as effective and well tolerated in the elderly as in younger patients, although the definition of “elderly” varies among trials, with each trial defining elderly as either 65, 70, or 75 years or older.^11^, ^12^, ^13^, ^14^ The results of the standard‐dose group in the present study did not display an increase in adverse events or a decrease in efficacy, consistent with the results of the MPACT trial or domestic phase I/II studies.^1^, ^5^

The present study was the first randomized Phase II trial to prospectively evaluate the efficacy of an attenuated regimen for elderly patients with metastatic pancreatic cancer. The attenuated regimen with extremely reduced amounts of GEM in the low‐dose group decreased the skipped doses and reduction rates, which might lead to similar efficacy as that observed in the standard‐dose group. Unexpectedly, this low‐dose GnP regimen was not beneficial in terms of the incident rates of adverse events. One possible reason for this could be the higher relative dose intensity of nab‐PTX, which was achieved by the lower skipped doses and reduction rates in the low‐dose group than in the standard‐dose group as shown in Table 2. These results suggest that an attenuated GnP regimen with an adjusted dosing schedule such as biweekly, rather than a reduced dose of GEM, may be useful to improve safety while ensuring the efficacy of chemotherapy. Hirao *et al*. conducted a randomized Phase II study comparing 4‐week and 3‐week schedules of GEM monotherapy in 90 patients with advanced pancreatic cancer. For the 4‐week and 3‐week schedules, the response rates (14.2% *vs* 17.1%), median progression‐free survival (112 days *vs* 114 days), and median overall survival (206 days *vs* 250 days) were comparable. Among adverse events, the incidence rate of thrombocytopenia was significantly lower for the 3‐week schedules (4.4%) than for the 4‐week schedule (26.6%).^15^ On the contrary, in a non‐inferiority study of S‐1 alternate‐day therapy *versus* S‐1 daily therapy in 190 patients with advanced pancreatic cancer, the median overall survival was 9.4 months in the alternate‐day group and 10.4 months in the daily group (hazard ratio; 1.19); therefore, the non‐inferiority of alternate‐day administration was not demonstrated.^16^ In addition, there was only a slight improvement in safety with the alternate‐day treatment, leading to the conclusion that the standard daily regimen should be recommended when using S‐1 therapy for advanced pancreatic cancer. Thus, the efficacy and safety of attenuated regimens for advanced pancreatic cancer appear to vary from regimen to regimen, and which regimen is more appropriate remains unclear, especially for elderly patients. To solve this issue, further randomized controlled trials are needed to evaluate various attenuated regimens in elderly patients with advanced pancreatic cancer.

The present study had several limitations. First, it was a Phase II study with response rate as the primary endpoint and a small number of cases. The lower overall survival in the low‐dose group, although not significant as shown in Table 3, might be due to the number of cases. Second, a survey of quality of life was not conducted in the present study. Higher quality of life might be seen in the elderly patients in the low‐dose GnP group than in the standard‐dose GnP group, and thus it remains possible that detailed data on tolerability would have produced differences between the two groups. Third, in light of the aging society, it is necessary to reconsider the definition of “elderly” in future clinical trials of pancreatic cancer. Finally, there was no advantage of low‐dose GnP other than cost efficiency. Therefore, a phase III trial with low‐dose GnP as one of the treatment arms should not be conducted. In conclusion, the present results show that the response rate and progression‐free survival of low‐dose GnP for metastatic pancreatic cancer in the elderly were comparable to those of conventional therapy; however, the low‐dose regimen did not reduce the rate of adverse events. Therefore, low‐dose GnP is not strongly recommended for elderly patients with metastatic pancreatic cancer.

## Data Availability

The datasets used and/or analyzed during the current study are available from the corresponding author on reasonable request.
